# The role of artificial intelligence in personal development and coaching among adolescents: a cross-sectional study and literature review

**DOI:** 10.1192/j.eurpsy.2026.11111

**Published:** 2026-07-31

**Authors:** F. Zaouali, I. Anes

**Affiliations:** 1 Emergency department and mobile emergency and resuscitation service, Tahar Sfar University Hospital, Mahdia; 2 Outpatient psychiatric consultation, Hadj Ali Soua Regional Hospital, Ksar Hellal, Monastir, Tunisia

## Abstract

**Introduction:**

Artificial intelligence (AI) is increasingly integrated into personal development and coaching platforms, offering personalized support in goal-setting, motivation, and emotional regulation. Adolescents represent a demographic particularly receptive to digital innovations, yet little is known about how AI-driven tools influence their personal growth and self-improvement efforts.

**Objectives:**

To assess knowledge, usage patterns, and attitudes toward AI-powered personal development tools and coaching applications among adolescents, and to synthesize current evidence on AI’s impact in this domain.

**Methods:**

A cross-sectional survey was conducted among 56 high school students aged 15 to 18 years. The questionnaire explored AI awareness, frequency of use of AI coaching tools (e.g., chatbots, personalized apps), perceived benefits and limitations, and willingness to adopt such technologies. Concurrently, a narrative literature review was performed using PubMed, PsycINFO, and Google Scholar with keywords including “artificial intelligence”, “personal development”, “coaching”, and “adolescents”. Studies published from 2010 to 2024 in English and French were screened, with 28 relevant articles retained for synthesis.

**Results:**

Among participants, 64% reported awareness of AI-based coaching tools, while 38% had actively used at least one such application. Commonly cited benefits included enhanced motivation, personalized feedback, and increased self-reflection. However, concerns about data privacy, lack of human empathy, and algorithmic bias were frequently mentioned. The literature review corroborated these findings, highlighting the potential of AI to democratize access to coaching and facilitate scalable interventions tailored to individual needs. Studies also emphasized limitations, such as the current inability of AI to fully replicate human emotional intelligence and the need for hybrid models integrating AI with human support. Overall, both empirical data and literature suggest a cautiously optimistic view of AI in adolescent personal development.

**Conclusions:**

Artificial intelligence holds significant promise in augmenting personal development and coaching for adolescents, fostering engagement and individualized guidance. Nonetheless, ethical considerations and the irreplaceable value of human interaction warrant balanced integration. Future research should focus on optimizing AI-human partnerships and evaluating long-term outcomes in youth populations.

**Disclosure of Interest:**

None Declared

